# Machine learning guided postnatal gestational age assessment using new-born screening metabolomic data in South Asia and sub-Saharan Africa

**DOI:** 10.1186/s12884-021-04067-y

**Published:** 2021-09-07

**Authors:** Sunil Sazawal, Kelli K. Ryckman, Sayan Das, Rasheda Khanam, Imran Nisar, Elizabeth Jasper, Arup Dutta, Sayedur Rahman, Usma Mehmood, Bruce Bedell, Saikat Deb, Nabidul Haque Chowdhury, Amina Barkat, Harshita Mittal, Salahuddin Ahmed, Farah Khalid, Rubhana Raqib, Alexander Manu, Sachiyo Yoshida, Muhammad Ilyas, Ambreen Nizar, Said Mohammed Ali, Abdullah H. Baqui, Fyezah Jehan, Usha Dhingra, Rajiv Bahl

**Affiliations:** 1 Center for Public Health Kinetics, Global Division, 214 A, LGL Vinoba Puri, Lajpat Nagar II, New Delhi, India; 2 College of Public Health, Department of Epidemiology, University of Iowa, 145 N. Riverside Dr. , S435, Iowa City, IA 52242 USA; 3 Department of International Health, Johns Hopkins Bloomberg School for Public Health, 615 N. Wolfe Street, Baltimore, MD 21205 USA; 4 Department of Paediatrics and Child Health, Aga Khan University, Karachi, Sindh Pakistan; 5 Projahnmo Research Foundation, Abanti, Flat # 5B, House # 37, Road # 27, Banani, Dhaka, 1213 Bangladesh; 6 Public Health Laboratory-IDC, Chake Chake, Pemba Tanzania; 7 International Centre for Diarrhoeal Disease Research, Mohakhali, Dhaka, 1212 Bangladesh; 8 Department of Maternal, Newborn, Child and Adolescent Health and Ageing, Avenue Appia 20, 1211 Geneva, Switzerland

**Keywords:** Pre-term births, Machine learning, Gestational age, New born screening

## Abstract

**Background:**

Babies born early and/or small for gestational age in Low and Middle-income countries (LMICs) contribute substantially to global neonatal and infant mortality. Tracking this metric is critical at a population level for informed policy, advocacy, resources allocation and program evaluation and at an individual level for targeted care. Early prenatal ultrasound examination is not available in these settings, gestational age (GA) is estimated using new-born assessment, last menstrual period (LMP) recalls and birth weight, which are unreliable. Algorithms in developed settings, using metabolic screen data, provided GA estimates within 1–2 weeks of ultrasonography-based GA. We sought to leverage machine learning algorithms to improve accuracy and applicability of this approach to LMICs settings.

**Methods:**

This study uses data from AMANHI-ACT, a prospective pregnancy cohorts in Asia and Africa where early pregnancy ultrasonography estimated GA and birth weight are available and metabolite screening data in a subset of 1318 new-borns were also available. We utilized this opportunity to develop machine learning (ML) algorithms. Random Forest Regressor was used where data was randomly split into model-building and model-testing dataset. Mean absolute error (MAE) and root mean square error (RMSE) were used to evaluate performance. Bootstrap procedures were used to estimate confidence intervals (CI) for RMSE and MAE. For pre-term birth identification ROC analysis with bootstrap and exact estimation of CI for area under curve (AUC) were performed.

**Results:**

Overall model estimated GA had MAE of 5.2 days (95% CI 4.6–6.8), which was similar to performance in SGA, MAE 5.3 days (95% CI 4.6–6.2). GA was correctly estimated to within 1 week for 85.21% (95% CI 72.31–94.65). For preterm birth classification, AUC in ROC analysis was 98.1% (95% CI 96.0–99.0; *p* < 0.001). This model performed better than Iowa regression, AUC Difference 14.4% (95% CI 5–23.7; *p* = 0.002).

**Conclusions:**

Machine learning algorithms and models applied to metabolomic gestational age dating offer a ladder of opportunity for providing accurate population-level gestational age estimates in LMICs settings. These findings also point to an opportunity for investigation of region-specific models, more focused feasible analyte models, and broad untargeted metabolome investigation.

**Supplementary Information:**

The online version contains supplementary material available at 10.1186/s12884-021-04067-y.

## Background

Of 15 million preterm births annually, 90% happen in Low and Middle Income Countries (LMICs) [1, 2] contributing to 1 million deaths < 5 years, 35% of deaths < 28 days [3]. Further 23.3 million infants (19.3% of live births) are born small for gestational age (SGA) in LMICs. Reduction by 10.0% in these would reduce neonatal deaths by 254,600 deaths [4]. Identifying and tracking this metric is therefore critical for advocacy, surveillance, research, evaluation of preventive strategies, and care of these high risk infants in LMICs. These in turn are essential to achieving United Nations Sustainable Development Goal 3 target 3.25 [2, 5](elimination of preventable under-five deaths by 2030).

The estimation of accurate gestational age at birth is essential for identifying both preterm and SGA births. Early ultrasonography examination, considered as gold standard for gestational age (GA) assessment is unavailable due to high equipment cost and lack of trained manpower in most LMICs settings. Recall of last menstrual period (LMP) [6] used in these settings is unreliable in estimation of GA [7]. Postnatal methods, birth weight and standardized scoring system (Dubowitz or Ballard scales) have poor reliability and high inter user variability limiting their usage [8–11].

In global health a need for novel tools is required that could help monitor these metrics in LMIC countries on a population scale [12]. Algorithms developed in three North American settings using routine metabolic screen data to derive GA estimates, have been shown to provide accurate estimates to within 1–2 weeks of ultrasonography basedGA [13–15]. Limited data for external validity of these methods in LMIC populations [16, 17] demonstrated satisfactory performance but lower accuracy for GA predictions especially among SGA new-borns in Africa and Asia. Reported publications used conventional statistical modelling approaches like linear/logistic regression and discriminant analysis. These statistical methods mainly focus on inference from fitting of a project-specific probability model [18]. Recent advances in Machine Learning (ML) techniques and big data analysis, allows for efficient handling of large numbers of predictors while incorporating non-linear association and complex interactions. ML techniques are more robust in nature and they mainly deal with the prediction of outcomes by using general-purpose learning algorithms to find patterns in a dataset without assumptions needed for conventional modelling. Recently, ML approaches has been shown to help preterm identification in hospital setting [19].

We hypothesized that application of ML to metabolite profile datasets of Alliance for Maternal and New-born Health Improvement (AMANHI) All children thrive (ACT) cohorts (representing both South Asia and Sub-Saharan Africa) would potentially improve the prediction of GA as compared to conventional approaches previously reported. The rationale for these cohorts and associated bio-bank procedures, and cohort characteristics have been described in previous publications [20, 21]. One of the objectives of the AMANHI study was to develop and validate programmatically feasible approaches to accurately assess the gestational age of babies after they are born. Additionally this method will not be dependent on the North American population datasets for generation of equation coefficients and enable regional adjustments in the future. Among various ML classifiers, we chose to use random forest as particularly well suited for clinical predictions [22]. We report the performance of our machine learning based GA estimation algorithms.

## Methods

### Study population

This study was undertaken using data from the Alliance for Maternal and New-born Health Improvement (AMANHI) All children thrive (ACT), community based, prospective pregnancy and New-born cohorts from Pemba (Tanzania), Sylhet (Bangladesh) and Karachi (Pakistan). These studies received ethical approval from the local and institutional ethics committees of all the three sites: ICDDR, B and John Hopkins University for Bangladesh, Aga Khan University for Pakistan and ZAMREC for Tanzania. The protocols were also approved by the Ethical Review Committee (ERC) at World Health Organization (WHO). Briefly, women were enrolled in early pregnancy and followed through delivery and the postpartum period (supplementary Fig. 1). All pregnant women were eligible to participate in the study. Written informed consent was obtained from all participants prior to study enrolment for collection of maternal and new-born data and samples. GA was established by ultrasonography [23] at screening using the fetal crown rump length (if < 14 weeks gestation) [24] or biparietal diameter and femur length (if ≥14 weeks) [23, 24]. All fetal biometry measurements were measured twice and then averaged for gestational age calculations [23, 25]. Birth weight (5 g sensitivity) was measured using standard new-born weighing scale (SECA corporation, Columbia, MD).

### Sample collection and processing

**Fig. 1:**
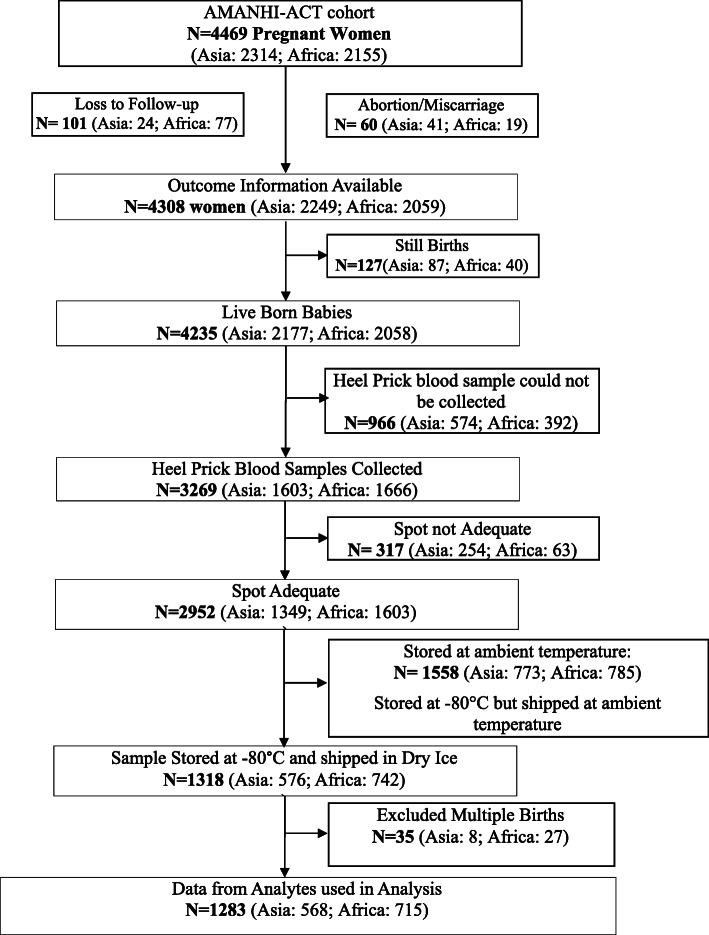
Consort cohort flow diagram

The metabolic screening data from 1283 samples used for this analysis was generated as part of the AMANHI collaboration with Department of Epidemiology, College of Public Health, University of Iowa, for evaluating external validity of the GA estimation methods developed based on American samples [13, 17]. An overview is provided in consort flow diagram (Fig. 1) and protocol process flow (supplementary Fig. 1). Heel prick blood spots were obtained on a protein saver card (Whatman^R^ 903, GE healthcare, USA), within 24–72 h of birth from new-born’s as per standard procedures. All 903 cards were labelled with barcoded unique identifier (ID’s), were air-dried and stored in air tight Ziplock bags with desiccant at − 80 °C. The 903 cards were shipped in dry ice to the University of Iowa where they were examined for quality. Then samples were sent to State Hygienic Laboratory, Ankeny, Iowa, USA at regular intervals (ensuring processing before potency window). Sixty-six metabolites (Supplementary Table 4) which included amino acid, acylcarnitines, enzymes and hormones were analysed using tandem mass spectrometry. Only singleton births (women who gave birth to only one child during a delivery, excluding twins, triplets) were included in the final analysis since analyte values are associated with birth status [26]. We excluded 35 non-singleton pregnancies in the final analysis.

### Selection of the machine learning algorithm

For implementing the machine learning algorithms, the dataset was divided into training and test datasets. This selection was made without replacement. Training dataset used was derived by combining stratified sampling of 80% from Africa and Asia separately. A K fold cross validation method was used as resampling procedure to evaluate the machine learning algorithms which effectively performed a 10 fold cross validation, with three repeats [27]. Performance of the models was assessed by comparing RMSE and MAE values on the training dataset. Four ML algorithms considered appropriate for this analysis; artificial neural network (ANN), decision tree (DT), support vector machines (SVM) and random forest (RF) were evaluated and compared [28]. Amongst these RF regressor performed better and was selected for testing on test dataset (results provided in Supplementary Table 1).

### Architecture of random Forest Regressor

ML models were generated using metabolite profiles along with birth weight and gender. RF regressor model for this analysis was built using 1) bootstrap sampling -with replacement and 2) Random feature –m = 10 in RF regressor. In this generation procedure was repeated until 10 decision trees were created to form a randomly generated “Forest”. We denoted the hyper-parameters for RF regressor as “mtry” (a variable imputed in the model) at each split node for performing regression; the default value of “mtry” is p/3 where p is the number of predictors [29]. Random forest is an algorithm built by multiple decision trees. So it is important to choose the hyper-parameter as it defines how each tree will be built.

### Implementation of random Forest Regressor

Sklearn.ensemble [30] (Random Forest Regressor package), a Python module was used for running the RF regressor. NumPy, Scipy and Pandas were used as python dependencies for running the module. R coding was used to create train and test datasets.

### Models used for the analysis

Four different models were used for the prediction of Gestational Age. (Supplementary Table 3; details of variables in the models provided in Supplementary Table 4).

**Model 1**: only one variable per metabolite (for all 66 metabolites) from the profile of the blood metabolites along with birthweight and gender.

**Model 2:** was designed to replicate model published by Ryckman et al. [13] which included the linear, squared and cubic values of the metabolites as predictors.

**Model 3:** was designed to replicate the model published by Wilson et al. 2017 [15] which included the linear, squared and cubic values of the metabolites along with birthweight and gender as predictors.

**Model 4 (selected Model):** Contained all the predictors used in the above three models.

### Best model selection

The best model was selected on the basis of root mean square error (RMSE) and mean absolute error (MAE).

The RMSE of a predicted model with respect to the estimated variable x_model_ has been defined as the square root of the mean squared error [31].

$$ \mathrm{RMSE}=\sqrt{\frac{\sum_{\mathrm{i}=1}^n{\left({X}_{obs,i}\hbox{-} {X}_{model,i}\right)}^2}{n}} $$

Where, x_obs_ is observed values, x_model_ is modelled values at time i.

Mean absolute error (MAE) has been calculated as

$$ \mathrm{MAE}=\frac{\sum_{\mathrm{i}=1}^n\left|{y}_i-{\chi}_i\right|}{n}=\frac{\sum_{\mathrm{i}=1}^n\left|{e}_i\right|}{n} $$

where x_i_ is the prediction and y_i_ is the true value.

### Confidence intervals for RMSE and MAE

Efficient computation of RMSE, MAE values and 95% confidence interval were estimated using bootstrapped procedures [32] (Python package, (Bootstrapped0.0.2) https://pypi.org/project/bootstrapped/) and for t with a fixed seed number of 1 using boot [33] and metrics packages in R.

### ROC analysis for evaluating discriminatory ability of the ML based GA

For ROC analysis we used Stata 16.1 (Stata Corp LLC, Texas USA) and MedCalc (MedCalc Software Ltd. Belgium). The predictions of the continuous outcome (predicted GA) were dichotomized to < 37 weeks (preterm birth) and > =37 weeks (term birth) for carrying out ROC analysis. Generation of ROC curve and AUC estimation was performed and interpreted using standard methods [34, 35]. We estimated Youden index J [36]

$$ J=\max \left\{ sensitivityf\left[c\right]+ specificityf\left[c\right]-1\right\} $$

where c ranges over all possible criterion values. Graphically, J is the maximum vertical distance between the ROC curve and the diagonal line. Bootstrapped 95% CI for Youden index and its corresponding criterion value were estimated [37, 38]. 95% CI for sensitivities and specificities were also estimated for a range of fixed and pre-specified sensitivities/specificities [33] and 95% CI estimated using bootstrapping [37, 38]. Comparison of ROC curves estimating difference, confidence interval and *p*-value were also performed using bootstrap methods [39, 40]. For the Bootstrap estimation a fixed seed was used to enable replication of the analysis.

## Results

### General characteristic of the cohort

Data from all 1318 new-borns having new-born metabolic screen analytes were included in the current analysis. Of these 742 samples were from Africa and 576 from Asia (Pakistan and Bangladesh). Baseline characteristics of the sample are provided in Table 1. The distribution of male and female subjects in the cohort was almost in the ratio of 1:1. The mean GA as confirmed by ultrasound was 38.5 ± 1.68 weeks (mean ± SD). Sample included 153 (11.6%) preterm births, 199 (15.1%) low birth weight and 271 (20.6%) SGA new-borns. Birth weight in African new-borns (3240.75 ± 585.88 g) tended to be higher than Asian new-borns (2774.55 ± 513.99 g).

**Table 1:** Cohort Characteristics Of Infants Included In The Metabolic Screening Study

Heel Prick Samples	All sites Combined (Total cohort)	Asia (Pakistan and Bangladesh)	Africa (Tanzania)
	***N*** = 1318	***N*** = 576	***N*** = 742
Gender			
Male	695 (52.7%)	268 (46.5%)	428 (57.6%)
Female	623 (47.3%)	308 (53.5%)	315 (42.3%)
Gestational Age Mean + S.D	38.53 + 1.68	38.35 + 1.67	38.68 + 1.68
> 37 weeks	1165 (88.4%)	492 (85.4%)	673 (90.7%)
< 37 weeks	153 (11.6%)	85 (14.6%)	69 (9.3%)
34–37 weeks	126 (82.4%)	71 (83.5%)	54 (78.3%)
< 34 weeks	27 (17.6%)	14 (16.5%)	15 (21.7%)
Birthweight (Mean + S.D)	3037.21 + 601.67	2774.55 + 513.99	3240.75 + 585.88
Birth Weight Category, n(%)			
≤ 2500 g	199 (15.1%)	153 (26.6%)	46 (6.2%)
> 2500 g	1119 (84.9%)	423 (73.4%)	696 (83.8%)
SGA Status			
Yes	272 (20.6%)	91 (15.8%)	181 (24.4%)
Multiple Birth Status	35 (2.7%)	8 (1.4%)	27 (3.6%)
Newborn Sample Collected (Hrs), Mean ± SD	49.0 ± 16.2	52.1 ± 19.4	46.6 ± 12.7

### Comparison of performance of gestational age estimation models

Initially we evaluated 4 models for performance predicting gestational age (Supplementary Table 4), the model 1 with only base terms for analytes was least accurate RMSE 1.38, model 2 using variables in final Iowa regression model [13] had RMSE of 1.29 and model 3 using variables in Ontario regression model [14] had RMSE 1.20 (Supplementary Table 2), the final all-inclusive model providing a RMSE of 1.02 (95% CI 0.91–1.14) was selected and evaluated further. For identification of preterm births, AUC of Model 4 was significantly better than model 1 [3.6% (95%CI − 1.2 to 8.5; *p* = 0.014)] and Model 2 [2.5% (95% CI − 2.2 to 7.3; *p* = 0.03)] (Supplementary Fig. 2).

Overall model estimated gestational age had a mean absolute error (MAE) of 5.2 days (95% CI 4.5–6.8), compared to gold standard ultrasound dating. Accuracy was slightly lower in Africa MAE 5.3 days (95% CI 54.8–6.2) than Asia MAE 5 days (95% CI 4.3–6.2) (Table 2). Contrary to the results from external validity of regression models [15, 17], performance in SGA new-borns was not appreciably reduced, MAE 5.3 days (95% CI 4.6–6.2 days). GA was correctly estimated to within 1 week of ultrasound-assigned values for 85.21% (95% CI 72.31–94.65) overall, 83.2% (95% CI 78.31–90.05) in African and 87.7% (95% CI 76.63–95.39) in Asian new-borns. Estimations performed as well in SGA new-borns within 1 week 83.9% (95% CI 71.21–92.32) (Table 2).

**Table 2:** Mean Abs Error and RMSE in weeks in final machine learning model

STATISTICS	Cohort		Africa		Asia	
	Overall	SGA	Overall	SGA	Overall	SGA
**Training Dataset**	**Test Data Set- Pooled **remaining		**Test Data Set -** remaining		**Test Data Set-** remaining	
**80% Pemba Samples + 80% Asian samples**	20% PembaSamples +	20% Asiansamples	20% Pemba Samples		20% Asian Samples	
MAE (95% CI)*	0.74 (0.65–0.98)	0.76 (0.65–0.88)	0.75 (0.61–0.89)	0.88 (0.75–1.16)	0.72 (0.62–0.88)	0.73 (0.61–0.95)
RMSE(95% CI)*	1.02 (0.91–1.14)	1.05 (0.91–1.19)	1.04 (0.89–1.16)	1.20 (1.10–1.31)	1.00 (0.89–1.16)	1.01 (0.93–1.19)
1 week difference (%)*	85.21 (72.31–94.65)	83.9 (71.21–92.32)	83.21 (78.31–90.05)	72 (65.67–79.34)	87.71 (76.63–95.39)	87.09 (77.67–94.21)
2 weeks difference (%)*	99.61 (91.42–100)	98.31 (89.74–100)	100 (93.32–100)	100 (92–79-100)	99.12 (91.56–100)	99.15 (90.45–100)
**Training Dataset 80% Africa samples for Africa and 80% Asia samples for Asia**			**Test Dataset (20% Africa samples)**		**Test Dataset (20% Asia samples**	
MAE (95% CI)*			0.71 (0.58–0.85)	0.83 (0.71–1.10)	0.68 (0.58–0.87)	0.71 (0.62–0.83)
RMSE (95% CI)*			0.96 (0.82–1.07)	1.13 (1.01–1.27)	0.93 (0.82–01.05)	0.97 (0.84–1.08)

*Bootstrapped,

*Detailed description of the analytes used in the models have been given in supplementary information

To evaluate impact of using a regionally trained algorithm (an important future prospect), we repeated the analysis with machine learning being trained by African sample for Africa estimations and Asian sample for Asia estimations. The model performance in spite of reduced samples for training improved both for Africa and Asia. The precision of MAE improved rather being reduced for Africa to 5 days (95% CI 4.1–6.0) and Asia to 4.8 days (95% CI 4.1–6.1) (Table 2). The same pattern was seen for RMSE (Table 2).

### Model discrimination of preterm birth

**Fig. 2:**
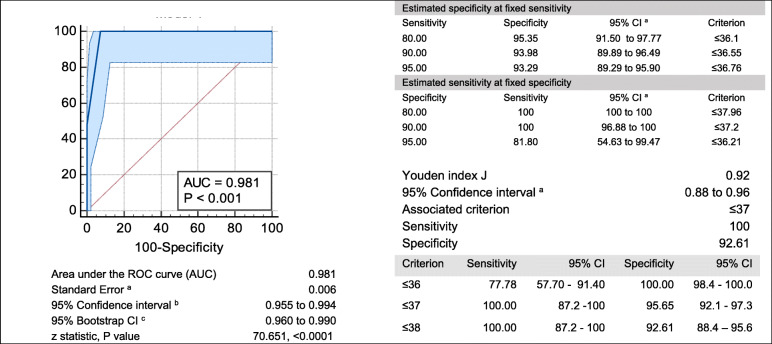
ROC analysis of Machine Learning Final Model in discrimination of gestation < 37 weeks

**Fig. 3:**
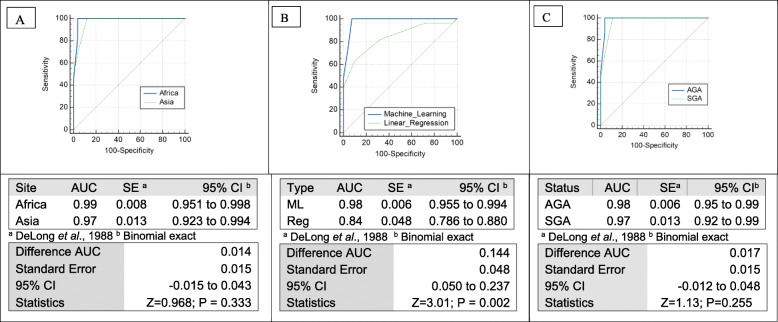
ROC analysis comparing Machine Learning performance. **A** By site. **B** With the estimates obtained from primary published regression analysis. **C** By SGA infants

For the ability to classify correctly preterm births (GA < 37 weeks), model in ROC analysis showed an area under curve (AUC) of 92.6% (BC 95% CI 87.5–96.1; *p* < 0.001). Criterion of ≥37 providing a sensitivity of 100% and specificity of 92.61% (Fig. 2). This model provided a significant improvement (difference in AUC 14.4% (95% CI 5.0–23.7; *p* = 0.002) over predicting GA by regression models in the same dataset, AUC 84% (95 CI 78.6–88.0), (Fig. 3b). There was no significant difference, 1.3% (95% CI − 1.5-4.3; *p* = 0.333) in AUC between Africa and Asia (Fig. 3a). The AUC between SGA and non-SGA new-borns also did not differ (AUC difference 1.7%;(95% CI − 1.2-4.8; *p* = 0.891) (Fig. 3c).

### Performance across gestational age categories

Estimation of RMSE and MAE as well as cross tabulation of actual and predicted GA by 2 weekly categories (Table 3), indicated that the accuracy of the current set of analytes was diminishing only at < 35.

**Table 3:**
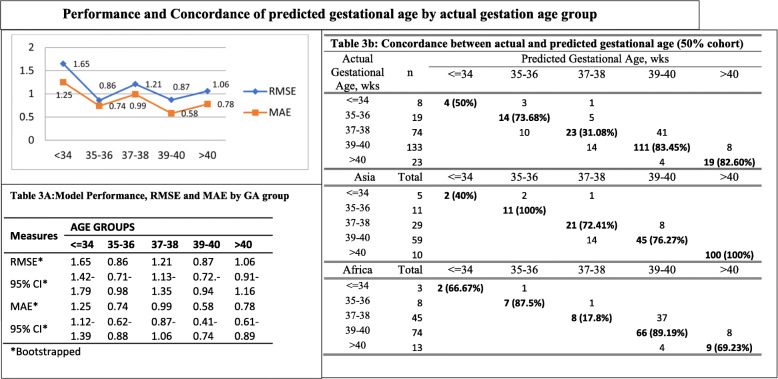
Performance and Concordance of predicted gestational age by actual gestation age group

## Discussion

This study has highlighted promising application of ML methodology to birth weight and new-born metabolomic screening data for improving postnatal prediction of gestation age at birth and discriminating between preterm and term new-borns. It also demonstrated ability of using LMICs data for training ML models and not needing external estimators from developed country datasets. In LMICs setting of South Asia and Sub-Saharan Africa, GA estimates from ML model were within an average of 5.2 days of ultrasonography based GA. The ML estimated GA enabled discrimination between pre-term and term births, AUC 98% was significantly better than regression estimated GA AUC 84%. The optimal criterion of ≤37 weeks providing a sensitivity of 100% and specificity of 92.616%.

As against lower performance of previous approaches [13, 15–17], in estimating GA in SGA sub population, our ML model estimates were within 5.3 days of ultrasonography based GA. This also reflected in the finding of a similar proportion with estimated gestation being within 1 week of the ultrasound confirmed gestation, 85.2% overall vs 83.9% in SGA subgroup. Use of data with 80% each of Asian and African data for training the models, was associated with some variation in predictive accuracy for Asia (average of 5 days) compared to Africa (average of 5.2 days). Using region specific data for training reduced the variation to 4.8 and 4.9 days respectively and improved the precision in spite of reduced sample size of training dataset (Table 2). Being preliminary proof of principle, these findings provide a vision for future implementations, where in region specific training datasets may improve global application of metabolomics based data for gestational age assessment.

Our study had a number of important strengths and also some limitations which need consideration while interpreting the results. The strengths included 1) a sampling frame which utilized samples from both South Asia and East Africa, home to most of the global mortality associated with preterm and SGA births, 2) the study design was nested in a well-described population-based cohort of pregnancy with WHO coordinated and harmonized protocols and SOP, 3) Active surveillance for early pregnancy identification with added measures (menstrual calendar, pregnancy), culminating in harmonized ultrasonography based gestation assessment between 8 and 19 weeks of gestation and 4) Sample collection, storage and shipment SOP based on pilot QC, resulting in high quality of samples. The primary limitation of this study is the participation bias against early preterm and early deaths before sample collection window. Relatively small proportion of actual births in this sub-sample limits our ability to comment on model performance in these sub-groups. Our finding of lower accuracy in pre-term ≤34 weeks may either reflect lack of association of the metabolites in that sub-group, a function of lack of sample in that group and/or bias introduced by selective exclusion of early deaths. Additionally, we were working with the limitation of a smaller sample size as compared to the usual sample sizes in machine learning universe. We did try to use methods appropriate to accommodating smaller sample sizes, however would not have been protected against extreme chance affecting the sample. The ability to train the model and precision of estimates is somewhat reassuring but would need confirmation.

Preterm births and SGA account for a substantial burden of mortality in first 5 years [3, 4]. Tracking these metrics is therefore critical for advocacy, allocation of resources, for surveillance, research, evaluation of preventive strategies, and care of these high-risk infants in low- and middle-income countries [3, 41]. At the core of this is the estimation of gestational age at birth and being able to discriminate pre-term births accurately. Difference in GA at birth of a week impacts neonatal morbidity, mortality, and long-term outcomes significantly [42, 43]. Our findings provide evidence that ML based gestational dating models improve upon the currently-used postnatal gestational age estimation methods [6, 8, 10, 11, 13–17]. However while considering implementation of metabolic gestational dating approaches for robust population-level estimates, current challenges and future opportunities that machine learning brings to this domain need consideration. Heel prick samples for new-born screening are typically collected at least 24 h after birth to accommodate postpartum fluctuations in analyte levels. This introduces a bias due to early deaths selectively occurring in pre-term births, further in LMICs settings as most mother-infant pairs do not stay in hospital beyond 24 after delivery [44]. In most LMICs new-born screening is not a standard practice and will entail challenges in sample collection and processing for metabolic screening, therefore scale up needs to include rethinking about development of cord-blood-specific models restricted to analytes less susceptible to fluctuations in the postnatal environment, establishing a profile of fewer selected metabolites that are measurable using less sophisticated equipment. While rethinking and investigating low-tech variations suitable to LMICs settings, also to consider are, newer high throughput trans proteome/metabolome platforms which are now becoming affordable (i.e. Seers Nano peptide technology [45]). An untargeted metabolomic approach may improve our ability to estimate GA postnatally while also identifying infants at risk of a variety of conditions. Use of a broader spectrum of analytes may also help select a restrictive model for cord blood. Building on this study, use of ML methodology would positively influence development of all the above approaches, due to flexibility, ability to use regional data for ML and not requiring circling back to accumulating large datasets with new intended analyte profiles.

## Summary

Towards implementing preterm birth surveillance initiatives [46] ML algorithms and models applied to metabolomic gestational age dating offer an opportunity ladder to provide accurate population-level gestational age estimates in LMICs settings. Further research should focus on application of ML enabling investigation and incorporation of region-specific models, evaluating broad untargeted metabolome or more focused feasible analyte pool with ML approaches. Derivation and optimization of cord blood metabolic profiles models predicting gestational age accurately would usher a new feasibility for use of this approach in LMICs settings.

## Supplementary Information

**Additional file 1: Fig. S1.** Pregnancy cohort and Study design. **Table S1.** Performance metrics of the different machine learning algorithms. **Table S2.** RMSE and Mean Abs Error in weeks obtained in three comparative models. **Fig. S2.** ROC analysis comparing discriminatory ability of four models evaluated. **Table S3.** Newborn screening metabolomic analytes estimated. **Table S4A.** Description of the model. **Table S4B.** Description of the model.

## Data Availability

The datasets used and/or analysed during the current study are available from the corresponding author on reasonable request.

## Abbreviations

LMICs: Low And Middle Income Countries
SGA: Small For Gestational Age
GA: Gestational Age
LMP: Last Menstrual Period
ML: Machine Learning
AMANHI: Alliance For Maternal And Newborn Health Improvement
ACT: All Children Thrive
ANN: Artificial Neural Network
DT: Decision Tree
RF: Random Forest
RMSE: Root Mean Square Error
MAE: Mean Absolute Error
CI: Confidence Interval
SD: Standard Deviation
WHO: World Health Organisation
QC: Quality Control
