# Supplementary material for: Machine learning guided postnatal gestational age assessment using new-born screening metabolomic data in South Asia and sub-Saharan Africa
Source: BMC Pregnancy Childbirth. 2021 Sep 7;21:609. doi: 10.1186/s12884-021-04067-y (PMC8424940; doi:10.1186/s12884-021-04067-y)
Supplement: Supplementary file 1 — Additional file 1: Fig. S1. Pregnancy cohort and Study design. Table S1. Performance metrics of the different machine learning algorithms. Table S2. RMSE and Mean Abs Error in weeks obtained in three comparative models. Fig. S2. ROC analysis comparing discriminatory ability of four models evaluated. Table S3. Newborn screening metabolomic analytes estimated. Table S4A. Description of the model. Table S4B. Description of the model. [file 12884_2021_4067_MOESM1_ESM.docx]

**Title: Machine learning guided postnatal gestational age assessment using new-born screening metabolomic data in South Asia and Sub-Saharan Africa**

**Running title: Gestational age estimation using Machine Learning**

Sunil Sazawal*^1^, Kelli K. Ryckman^3^*, Sayan Das^1*^, Rasheda Khanam^4^, Imran Nisar^5^, Elizabeth Jasper^3^, Arup Dutta^1^, Sayedur Rahman^6^, Usma Mehmood^5^, Bruce Bedell^3^, Saikat Deb^2^,Nabidul Haque Chowdhury^6^, Amina Barkat^5^, Harshita Mittal^1^, Salahuddin Ahmed^6^, Farah Khalid^5^, Rubhana Raqib^7^, Alexander Manu^8^, Sachiyo Yoshida^8^, Muhammad Ilyas^5^, Ambreen Nizar^5^,Said Mohammed Ali^2^, Abdullah H. Baqui^4^**, Fyezah Jehan^5^**, Usha Dhingra^1^**, Rajiv Bahl^8^**

*joint first author **joint last author

1. Center for Public Health Kinetics, Global Division, 214 A, LGL Vinoba Puri, Lajpat Nagar II, New Delhi, India
2. Public Health Laboratory-IDC, Chake Chake, Pemba,Tanzania
3. University of Iowa, College of Public Health, Department of Epidemiology, 145 N. Riverside Dr. , S435, Iowa City, IA 52242,
4. Department of International Health, Johns Hopkins Bloomberg School for Public Health, 615 N. Wolfe Street, Baltimore, Maryland 21205
5. Aga Khan University, Department of Paediatrics and Child Health, Karachi, Sindh, Pakistan
6. PROJAHNMO Research Foundation, Abanti, Flat # 5B & 5D, House # 37, Road # 27, Banani, Dhaka-1213, Bangladesh
7. International Center for Diarrheal Disease Research, Mohakhali, Dhaka 1212, Bangladesh
8. World Health Organization (MCA/MRD), Geneva, Switzerland

**Corresponding author**

**Sunil Sazawal PhD;**

Center for Public Health Kinetics, Global Division, 214 A, LGL Vinoba Puri, Lajpat Nagar II, New Delhi, India. E-mail; [ssazawal@jhu.edu](mailto:ssazawal@jhu.edu) ; Tel +91-11-41724902

**Rajiv Bahl MD;**

World Health Organization (MCA/MRD), Avenue Appia 20, 1211 Geneva, Switzerland: e-mail:[bahlr@who.int](mailto:bahlr@who.int), Tel: +41-22-7912111

**Supplementary Figure 1: Pregnancy cohort and Study design**


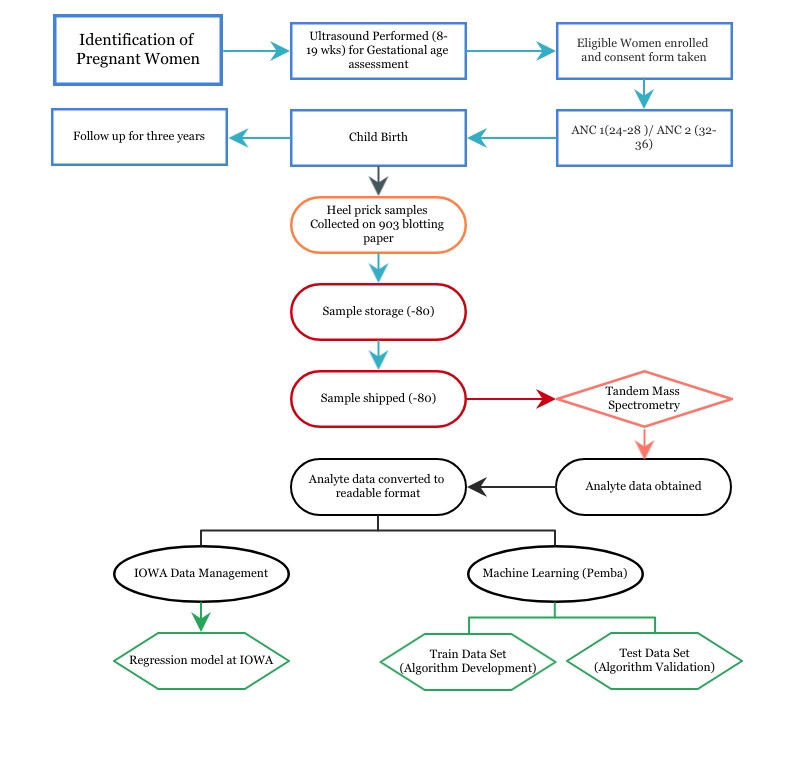


**Supplementary Table 1: Performance metrics of the different machine learning algorithms**

| **Models: Random Forest, Decision Tree, ANN, SVM** | | | | | | | | |
| --- | --- | --- | --- | --- | --- | --- | --- | --- |
| **Number of resample : 30** | | | | | | | | |
| **ALGORITHMS** | **RMSE** | | | | **MAE** | | | |
|  | **Min** | **Median** | **Mean** | **Max** | **Min** | **Median** | **Mean** | **Max** |
| *Random Forest* | 1.27 | 1.34 | 1.33 | 1.38 | 1.27 | 1.34 | 1.33 | 1.38 |
| *Decision Tree* | 1.3 | 1.37 | 1.36 | 1.4 | 1.3 | 1.37 | 1.36 | 1.4 |
| *ANN* | 1.33 | 1.38 | 1.39 | 1.46 | 1.33 | 1.38 | 1.39 | 1.46 |
| *SVM* | 1.35 | 1.43 | 1.42 | 1.49 | 1.35 | 1.43 | 1.42 | 1.49 |

**Supplementary Table 2: RMSE and Mean Abs Error in weeks obtained in three comparative models**

| ***Model 1. Using all analytes (our data) + birthweight + gender + removed all women with multiple births** | | | |
| --- | --- | --- | --- |
| **Training Dataset: 80% Pemba Samples + 80% Asian samples** | | **Mean Abs Error (95% C.I) (in weeks)** | **RMSE (95% C.I) (in weeks)** |
| *Test Data (1):* | Combined : 20% rem Pemba Samples + 20% rem Asian samples | 0.93 (0.81-1.07) | 1.38 (1.21-1.49) |
| *Test Data (2):* | Africa: 20% remaining Pemba Samples | 0.96 (0.87-1.08) | 1.39 (1.23-1.51) |
| *Test Data (3):* | Asia: 20% remaining Asian Samples | 0.91 (0.79-1.05) | 1.37 (1.19-1.54) |
| ***Model 2. Using Rykman*et al’s* analytes (linear,squared,cubic) + removed all women with multiple births** | | | |
| **Training Dataset: 80% Pemba Samples + 80% Asian samples** | | **Mean Abs Error (95% C.I) (in weeks)** | **RMSE (95% C.I) (in weeks)** |
| *Test Data (1):* | Combined : 20% rem Pemba Samples + 20% rem Asian samples | 0.87 (0.78-1.03) | 1.29 (1.18-1.41) |
| *Test Data (2):* | Africa: 20% remaining Pemba Samples | 0.85 (0.75-1.01) | 1.27 (1.15-1.38) |
| *Test Data (3):* | Asia: 20% remaining Asian Samples | 0.89 (0.80-1.06) | 1.32 (1.22-1.45) |
| ***Model 3. Using Murphy *et al’s* (linear + squared + cubic + combinations + gender + birthweight + removed all women with multiple birth status** | | | |
| **Training Dataset: 80% Pemba Samples + 80% Asian samples** | | **Mean Abs Error (95% C.I) (in weeks)** | **RMSE (95% C.I) (in weeks)** |
| *Test Data (1):* | Combined : 20% rem Pemba Samples + 20% rem Asian samples | 0.84 (0.72-1.01) | 1.20 (1.09-1.31) |
| *Test Data (2):* | Africa: 20% remaining Pemba Samples | 0.87 (0.79-1.08) | 1.23 (1.12-1.35) |
| *Test Data (3):* | Asia: 20% remaining Asian Samples | 0.81 (0.67-0.97) | 1.17 (1.08-1.28) |

**Supplementary Figure 2: ROC analysis comparing discriminatory ability of four models evaluated**

| **** | \| Model_4 ~ Model_1 \| \| \| --- \| --- \| \| Difference between areas \| 0.0365 \| \| Standard Error ^a^ \| 0.0252 \| \| 95% Confidence Interval \| -0.0128 to 0.0858 \| \| z statistic; P value \| 1.450; P = 0.014 \| \| Model_4 ~ Model_2 \| \| \| Difference between areas \| 0.0254 \| \| Standard Error ^a^ \| 0.0246 \| \| 95% Confidence Interval \| -0.0228 to 0.0737 \| \| z statistic; P value \| 1.034; P = 0.034 \| \| Model_4 ~ Model_3 \| \| \| Difference between areas \| 0.00564 \| \| Standard Error ^a^ \| 0.00846 \| \| 95% Confidence Interval \| -0.0109 to 0.0222 \| \| z statistic; P value \| 0.667; P = 0.5051 \|   ^a^ DeLong *et al*., 1988, ^b^ Binomial exact |
| --- | --- | --- | --- | --- | --- | --- | --- | --- | --- | --- | --- | --- | --- | --- | --- | --- | --- | --- | --- | --- | --- | --- | --- | --- | --- | --- | --- | --- | --- | --- | --- |

| Model | AUC | SE | 95% CI |
| --- | --- | --- | --- |
| Model_3 | 0.975 | 0.00814 | 0.947 to 0.990 |
| Model_4 | 0.981 | 0.00680 | 0.955 to 0.994 |

| Model | AUC | SE | 95% CI |
| --- | --- | --- | --- |
| Model_1 | 0.944 | 0.0254 | 0.908 to 0.969 |
| Model_2 | 0.955 | 0.0249 | 0.922 to 0.977 |

**Supplementary Table 3: Newborn screening metabolomic analytes estimated**

| **Amino Acids** | Alanine, Arginine, Leucine, Methionine, Ornithine, Phenylalanine, Tyrosine, Valine, Citruline, Glutamate, Arginine:Onithine, Citruline:Arginine, Leucine:Alanine, Leucine:Phenylalanine, Methionine:Phenylalanine, Phenylalanine:Tyrosine, Tyrosine:Phenylalanine |
| --- | --- |
| **Acylcarnitines** | Carnitine (C0), C0:C16, C0:C18, C10, Decenoyl carnitine (C10:1), C12, C12:1, Tetradecanoylcarnitine (C14), Hydroxytetradecanoylcarnitine (C14:1OH), Tetradecenoyl carnitine (C14:1),C14:1:C12:1, C14:1:C16, C14:2, Palmitoylcarnitine (C16), 3 hydroxyhexadecenoyl carnitine (C16:1OH), Palmitoleylcarnitine (C16:1), 3- Hydroxypalmitoleylcarnitine (C16:1:1OH), C16:1OH:C16, Stearoyl carnitine (C18), C18:1OH, Oleoylcarnitine (C18:1), 3 -Hydroxyoleoylcarnitine (C18:1:1OH), Linoleoylcarnitine (C18:2), Acetylcarnitine (C2), Propionylcarnitine (C3), Malonylcarnitine (C3DC), C3C2, C4, Methylmalonylcarnitine (C4DC), C4:1OH, C4C2, C4C3, Isovalerylcarnitine + Methylbutyrylcarnitine (C5), Glutarylcarnitine (C5DC), C5DC:C16, C5DC:C8, C5:1OH, Tiglylcarnitine (C5:1), C5:C2, C5:C3, C6, Methylglutarylcarnitine (C6DC), Octanoylcarnitine (C8), Octenoylcarnitine (C8:1), C8:C10 |
| **Endocrine**  **markers** | Hydroxyprogestrone, Thyroid Stimulating Hormone |
| **Enzyme and**  **Coenzyme markers** | Galactose-1 phosphate uridylyl transferase, Succinyl lactone |

**Supplementary Table 4A: Description of the model**

**Supplementary Table 4B: Description of the model (…continued)**
